# Making the Connection: Polar Sites Are Key to Assembly in Many Immune Receptors

**DOI:** 10.1371/journal.pbio.0040168

**Published:** 2006-04-25

**Authors:** Mary Hoff

Your ability to survive a paper cut or conquer a cold depends on immune system cells that circulate through your body, carrying out search-and-destroy missions against bacteria, viruses, and anything else that's not you. Such cells bear invader-snagging transmembrane proteins called receptors. When a receptor latches onto an invader (or a cell overtaken by invaders), it sends a signal through an associated signaling module. The complex then transmits a “got one!” message to the interior of the cell, where it elicits a full-scale defensive response.

Proper assembly of receptors with the right signaling modules is key to an appropriate immune response, and incorrect assembly could well be a factor in immune system disorders such as chronic inflammation and autoimmunity. Jianwen Feng, Matthew E. Call, and Kai W. Wucherpfennig report fascinating findings about receptor assembly that shed light on how signaling modules can be versatile yet appropriately specific.

The researchers looked at the assembly of signaling modules with receptors from two key protein families, immunoglobulins and C-type lectins. The receptors they studied were KIR, NKG2D, NKG2C/CD94, and FcαRI. The signaling modules under study were DAP10, which assembles with only NKG2D; DAP12, which assembles with KIR and many other receptors; and Fcγ, which assembles with FcαRI and other receptors as well.

Previous research had shown that the assembly of receptors and signaling modules often involves attraction between one basic amino acid residue in the transmembrane part of the receptor and two acidic residues in the transmembrane part of the signaling module. The diversity of receptors that assemble with DAP12 and the wide variation among species in the amino acid makeup of DAP12 suggest that much of the rest of the transmembrane portion is less important for assembly. To test that, the researchers replaced all of the transmembrane residues in KIR (an immunoglobulin) with polyvaline or polyleucine except the one basic amino acid; they found it still assembled with DAP12 as long as the key acidic residues (both aspartic acid) of DAP12 had not been altered. If they replaced an aspartic acid, however, assembly was impaired. The authors also tested the altered KIR molecule in an actual cell and found that neither assembly of KIR nor its transport to the cell surface was prevented by the substitution.

Is the singular importance of the acid–base attraction for assembly true for other receptors as well? The researchers found that it is for the assembly of NKG2D, a C-type lectin, with DAP10. The same held true in the case of the assembly of the NKG2C portion of the NKG2C/CD94 receptor with DAP12 and for the assembly of FcαRI with Fcγ (although in the latter case, assembly was reduced).

Why, with the ubiquity of this assembly mechanism, don't receptors and signaling modules end up making inappropriate matches? The base used to make the connection, the researchers found, is one key. KIR uses lysine, while FcαRI and NKG2D use arginine. When the authors tried switching lysine for arginine or vice versa for KIR and FcαRI—or tried to get the signaling modules to associate with each other's receptor— assembly failed. (NKG2D assembly with DAP10 wasn't much affected, though, by switching out arginine and lysine.) Other tests showed that the position of the basic amino acid, the size and shape of the part of the receptor that sticks out of the cell membrane, and the different affinities of various signaling modules for various receptors also contribute to success in making the right receptor–signaling module match.

The bottom line? The mechanism by which immune system receptors and signaling modules assemble is similar enough among molecule types to allow some signaling modules to hook up with a wide range of receptors. At the same time, the specificity of the bases within the membrane, the shape of things above the membrane, and the differential ease of assembly of signaling modules provides needed specificity to prevent inappropriate assembly.

## 

**Figure:**
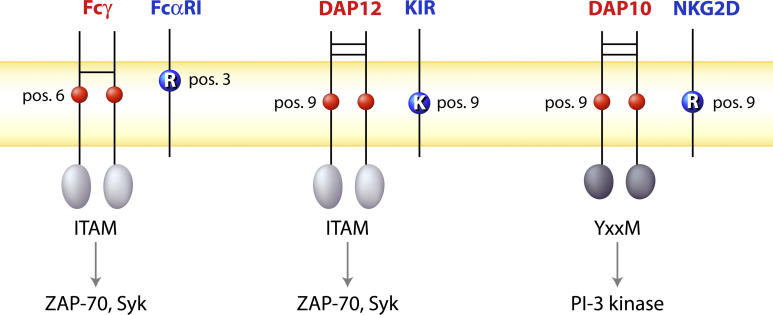
Many activating receptors in the immune system assemble with their dimeric signaling modules in the membrane through an interaction between their basic transmembrane residue and a pair of acidic transmembrane residues of the signaling module.

